# An artificial intelligence-based audio recognition system for dysarthria home-based rehabilitation: A proof-of-concept study

**DOI:** 10.1371/journal.pdig.0001794

**Published:** 2026-10-21

**Authors:** Vicente Sancenon, Pamela Y. S. Oh, Elvina K. W. Tay, Jarrod Z. X. Lee, Megan L. E. Soon, Han Leong Goh, Nicodemus Oey, Andy W. A. Ta

**Affiliations:** 1 Department of Future Technology & Innovation - Data, AI & Innovation, Synapxe, Singapore, Singapore; 2 Rehabilitation, Ng Teng Fong General Hospital and Jurong Community Hospital, National University Health System, Singapore, Singapore; 3 Department of Post-Acute & Continuing Care, Jurong Community Hospital, National University Health System, Singapore, Singapore; 4 Division of Rehabilitation Medicine, Department of Medicine, National University Hospital, National University Health System, Singapore, Singapore; 5 Lee Kong Chian School of Medicine, Nanyang Technological University, Singapore, Singapore; 6 Yong Loo Lin School of Medicine, National University of Singapore, Singapore, Singapore; National Research Center for Rehabilitation Technical Aids, CHINA

## Abstract

Dysarthria is a common motor speech disorder affecting individuals with neurological conditions, with high prevalence among stroke survivors. Effective treatment of dysarthria requires accurate assessment of severity and the provision of speech exercises tailored to the degree of impairment. However, traditional speech intervention faces several limitations, including limited accessibility to therapists and low treatment adherence rates. AI-driven solutions have the potential to address these limitations by automating the detection of dysarthria severity and recommending personalized exercises. Although substantial progress has been made in the automated detection and classification of dysarthria severity, the performance of these approaches on less controlled, real-world speech remains insufficiently characterized. This proof-of-concept study describes the development and evaluation of Whisper-ST1, a novel framework for classifying dysarthric speech severity from audio files by fine-tuning a pretrained automatic speech recognition model. Our framework builds on previous studies by incorporating real-world data alongside traditional research datasets, adapting a model architecture pretrained on a large corpus of audio data, applying a progressive unfreezing strategy to prevent catastrophic forgetting of relevant speech features while promoting flexible adaptation to the new task, and implementing an ordinal regression loss to minimize the impact of misclassification errors. Ablation experiments demonstrate the performance contributions of individual pipeline components in isolation. Whisper-ST1 achieves 79% [67% – 100%] per-speaker F1 in the classification of dysarthria severity when tested on real-world samples, and shows strong agreement with two speech therapists’ ratings of dysarthria (Weighted Cohen’s kappa of 0.814 [0.400 – 1.000] and 0.878 [0.647 – 1.000]), comparable to inter-therapist agreement (0.851 [0.550 – 1.000]). Model errors are predominantly confined to adjacent severity levels, suggesting preservation of ordinal structure. These findings demonstrate the feasibility of leveraging pretrained speech models for dysarthria severity classification in heterogeneous audio conditions. and provide a foundational step toward AI-assisted tools for remote monitoring and support of dysarthria rehabilitation.

## Author summary

Dysarthria is a motor speech disorder characterized by muscle problems which result in unclear or slurred speech. This disorder affects millions of people worldwide. Effective treatment requires intensive and frequent rehabilitation sessions with speech therapists, who provide personalized rehabilitation programs tailored to each individual dysarthria severity. However, speech therapy currently faces several major challenges, including high costs, low adherence rates, and limited therapist availability. These challenges are particularly pressing in Singapore, where an ageing population and a shrinking workforce necessitate innovative healthcare solutions. In this study, a multidisciplinary team of speech therapists, rehabilitation physicians, and data specialists developed Whisper-ST1, an Artificial Intelligence system that accurately assesses dysarthria severity from audio samples, closely matching expert therapists’ evaluations. Unlike previous work that relied solely on curated research data, we distinctively built Whisper-ST1 using both controlled clinical recordings and real-world speech samples, thus increasing the range of speech and recording conditions represented during model development. Whisper-ST1 classifies dysarthria severity at overall speaker level with 83% accuracy, 95% CI [58% – 100%], showing promising performance within this proof-of-concept study. With further clinical validation, the system may be suitable for integration into a mobile application to support remote, personalized speech therapy for individuals with dysarthria. Collectively, this work demonstrates the feasibility of AI-based dysarthria severity classification using speech recorded under diverse conditions.

## Introduction

Speech disorders affect millions of people worldwide, significantly impacting their quality of life and ability to communicate effectively. Among these disorders, dysarthria accounts for a significant proportion, with 52% of stroke survivors experiencing dysarthria [1]. A very common motor speech disorder which also increases with age, dysarthria results from damage to the central or peripheral nervous system, and can stem from various etiologies including stroke, Parkinson's disease, traumatic brain injury, and other neurodegenerative conditions [2,3]. The global aging population trend has intensified the demand for speech therapy services, particularly in countries like Singapore, where it is projected that one in four citizens will be above 65 years old by 2030 [4]. This demographic shift, coupled with a decreasing old-age support ratio, has placed healthcare resource allocation, including speech therapy services, at a critical junction. The surge in demand across various healthcare facilities, including acute hospitals, community hospitals, polyclinics, and outpatient day rehabilitation centers, has highlighted the pressing need for innovative solutions to complement traditional therapy methods.

Adequate treatment of dysarthria requires assessing its degree of severity and tailoring the rehabilitation program accordingly. Dysarthria severity assessments are either done formally with a standardized assessment or informally based on perceptual ratings. Speech intelligibility, articulatory precision, rate, and volume are the main features that are identified when assessing the level of speech impairment. While inter-rater reliability of dysarthria severity estimations is moderate to excellent, validity and reliability of the measures are variable between therapists [5]. Therapeutic methods are also fraught with inter- and intra-rater variability, poor measurement precision due to coarse ordinal scaling, and susceptibility to contextual bias [3,6–10]. Motor speech disorders like dysarthria require high frequency and intensive practice to achieve meaningful clinical outcomes, in accordance with principles of motor learning and neuroplasticity [11–13]. *Be Clear* is one of the more formalized and established speech protocols with efficacious results [14]. The protocol was based on the Lee Silverman Voice Treatment program, which has shown to improve volume in patients with Parkinson’s disease after 16 sessions (i.e., 4 sessions per week for 1 month), inadvertently improving their speech as well [15]. In real clinical settings, this procedure is challenging as the frequency of speech practice is often limited by therapists’ availability, costs, geographic access, and patient compliance. In home-based settings, patients frequently struggle with maintaining motivation, establishing routines, and receiving real-time feedback, which are crucial for effective rehabilitation [5]. Adherence to home-based speech-language therapy programs remains understudied, with limited evidence regarding strategies to improve and monitor adherence [16]. A study conducted in outpatient rehabilitation settings in Singapore found that low adherence rates were contributed by financial barriers, such as transport to and from clinics and the cumulative cost of frequent rehabilitation sessions over months [17]. With rising healthcare costs and manpower constraints, there is an urgent need for an effective solution that allows patients to practice dysarthria therapy on their own and achieve the same treatment gains as standard therapy with fewer speech therapist sessions.

The advent of digital technologies and artificial intelligence (AI) has opened new avenues for addressing these challenges. Automatic detection and severity classification of dysarthria from speech has evolved from traditional feature-engineered pipelines with machine learning classifiers to advanced self-supervised speech representations with deep learning architectures. Early work leveraged acoustic features such as MFCCs and prosodic measures with classifiers like SVMs, CNNs and RNNs, demonstrating the feasibility of severity prediction from speech signals [18,19]. More recent state-of-the-art methods leverage pre-trained models (such as wav2vec2.0, HuBERT, distilALHuBERT) to extract high-level embeddings in conjunction with machine learning or CNN classifiers, significantly outperforming traditional handcrafted features for both dysarthria detection and multi-class severity classification tasks [20,21]. Parallel research has demonstrated the efficacy of end-to-end architectures that convert audio signal into spectral and temporal patterns, including pure CNNs [22–24] or hybrid CNN-RNN architectures [25]. Recent approaches incorporate transformer-based architectures by applying attention-based classifiers to time-frequency representations [26]. In addition, current research also explores transfer learning techniques by fine-tuning CNNs pre-trained with a large image database [27] or self-supervised speech models pre-trained on large-scale healthy speech [28,29]. Notably, recent frameworks integrate speaker-agnostic regularisation techniques, such as multi-task learning, to improve generalization [28–30]. These approaches achieve high accuracy on benchmark datasets, such as UA-Speech [31] and TORGO corpora [32], reflecting a shift toward more robust, generalizable models for automated dysarthria assessment.

Despite these advances in automated dysarthria classification, current market offerings in digital speech therapy tools are limited and often fall short of providing real-time feedback, personalized guidance, and adaptive difficulty levels, reflecting a gap between the models performance in research settings and their clinical usefulness [33,34]. Existing solutions are generic and inflexible, failing to address the unique needs of individuals with neurological speech impairments.

Three key issues have been identified in the current landscape of AI-driven speech therapy solutions:

- Accuracy and generalization limitations due to insufficient comprehensive datasets of impaired speech samples.
- Lack of accurate feedback, tailored to the degree of severity of the disease, which is highly valued by users [35].
- Inability to detect subtle pronunciation errors and speech distortions unique to neurological impairments, as well as population-specific accent and pronunciation variability.

To address these gaps, we developed and evaluated a novel AI model to classify the severity of dysarthria speech using clinically objective labeling methods. Our study advances previous work on automated dysarthria speech recognition and severity classification in several distinct ways:

- Incorporated dysarthric speech data from social media platforms to increase the range of recording conditions represented in the development dataset
- Labeled the dataset using a quantifiable, reproducible, and clinically relevant rating methodology, rather than perceptual assessment.
- Designed a universal and scalable data partition strategy to preserve speaker and class balance across splits and mitigate potential model bias.
- Adapted a new model architecture for dysarthria severity classification by fine-tuning an Automatic Speech Recognition (ASR) model pretrained with a massive corpus of audio data.
- Implemented a regression objective to minimize the impact of misclassification errors for ordinal data during fine-tuning.
- Applied a progressive fine-tuning strategy in automated dysarthria speech detection by gradually unfreezing model layers.

## Results

### Classification metrics

To develop an AI system to classify dysarthria severity from audio files, a pretrained ASR model (Whisper-base) [36] was fine-tuned and adapted for a regression task (Methods). We call our model Whisper-ST1.

At sample level, Whisper-ST1 achieved an overall accuracy of 75% and a macro-F1 score of 72%, with a Precision of 81% and a Recall of 72% (Table 1). Performance was high in the normal/mild (93% F1 score) and moderate (82%) severity levels, and moderate in the severe (59%), and profound (52%) levels, indicating that Whisper-ST1 discriminates with high accuracy normal speech and moderate dysarthria, and with moderate accuracy severe and profound dysarthria. All misclassified samples deviated only by one severity level from the ground truth rating, showing that, in general, Whisper-ST1 captures severity progression correctly, with some errors in capturing boundary separation between contiguous levels, especially severe-profound (Table 2).

**Table 1:** Whisper-ST1 performance metrics.

	Sample level*			Speaker level*^,#^		
	Precision	Recall	F1	Precision	Recall	F1
Norm/Mild	1.00	0.87	0.93	1.00 [1.00 – 1.00] / 99.1%	1.00 [1.00 – 1.00] / 99.1%	1.00 [1.00 – 1.00] / 99.1%
Moderate	0.79	0.85	0.82	1.00 [1.00 – 1.00] / 96.2%	1.00 [1.00 – 1.00] / 96.2%	1.00 [1.00 – 1.00] / 96.2%
Severe	0.46	0.81	0.59	0.50 [0.00 – 1.00] / 99.1%	1.00 [1.00 – 1.00] / 88.6%	0.67 [0.33 – 1.00] / 66.7%
Profound	1.00	0.35	0.52	1.00 [1.00 – 1.00] / 64.3%	0.33 [0.00 – 1.00] / 96.8%	0.50 [0.33 – 1.00] / 64.3%
**Macro Average**	**0.81**	**0.72**	**0.72**	**0.88 [0.67 – 1.00]** / 100%	**0.83 [0.67 – 1.00]** / 100%	**0.79 [0.67 – 1.00]** / 100%
**Weighted Average**	**0.83**	**0.75**	**0.75**	**0.92 [0.81 – 1.00]** / 100%	**0.83 [0.58 – 1.00]** / 100%	**0.82 [0.64 – 1.00]** / 100%

*Overall accuracy: Per-sample: 75%; Per-speaker: 83% [58% – 100%] / 100%.

**^#^Per-speaker**: Point estimate [95% CI from 10,000 bootstrap samples] / % Valid bootstrap samples.

**Table 2:** Whisper-ST1 confusion matrices.

		Sample level				Speaker level			
		Predicted				Predicted			
Norm/Mild	Moderate	Severe	Profound	Norm/Mild	Moderate	Severe	Profound
Actual	Norm/Mild	20	3	0	0	4	0	0	0
	Moderate	0	23	4	0	0	3	0	0
	Severe	0	3	13	0	0	0	2	0
	Profound	0	0	11	6	0	0	2	1

From a practical perspective, it is more relevant to determine dysarthria severity at speaker level rather than at speech sample level. While per-sample metrics reflect model consistency across inter-sample variability, per-speaker metrics capture practical applicability more closely. Moreover, individual samples are not independent but clustered by speaker, as a single label is assigned to each speaker. Consequently, sample level metrics may overstate the effective sample size and statistical power. Therefore, speaker level metrics were computed and used as the primary endpoint of the analysis.

To assess speaker level performance, classification and agreement metrics were estimated by applying a sample voting scheme (Methods). However, the cohorts of the test set are relatively small (12 speakers for the classification metrics and 9 speakers for the agreement metrics), potentially biasing point estimates of speaker level metrics. To address this limitation, speaker level bootstrapping was performed to compute 95% Confidence Intervals (CIs) of the performance metrics estimates. Notably, at speaker level, Whisper-ST1 performance was consistent with sample level, achieving an overall accuracy of 83% [58% – 100%] and a macro-F1 score of 79% [67% - 100%], with a Precision of 88% [67% - 100%] and a Recall of 83% [67% - 100%] (Table 1). Performance was high in the normal/mild (100% F1 score [100% - 100%]), and moderate (100% [100% - 100%]) levels, and moderate in the severe (67% [33% - 100%]), and profound (50% [33% - 100%]) levels, in agreement with the results at sample level, indicating that Whisper-ST1 identifies normal/mild and moderate dysarthria with high accuracy but shows a moderate tendency to underscore profound dysarthria as severe. Out of 12 speakers, 10 were classified correctly and 2 profound cases were misclassified as severe, showing an overall acceptable performance with a moderate error rate in the higher severity range (Table 2). The lower bounds of the CIs are driven by Whisper-ST1 tendency to underrate profound dysarthria while the upper bounds reflect higher model accuracy in the classification of normal to moderate severity. Therefore, performance analyses at speaker level reveal that Whisper-ST1 is overall accurate in classifying dysarthria severity upon obtaining a consensus rating of multiple samples from the same speaker.

### Correlation analyses

At sample level, Whisper-ST1’s predictions displayed strong positive correlation with the ground truth labels (Tau = 0.789) slightly lower than the correlation of two independent speech therapists’ ratings with the ground truth (Tau = 0.884; Tau = 0.895) and between themselves (Tau = 0.846) (Table 3). Whisper-ST1 showed a mild tendency to underscore higher severity levels (profound, severe) and overscore lower severity levels (moderate, normal/mild) relative to the speech therapists’ scoring (Fig 1). These observations were confirmed at speaker level, where Whisper-ST1’s ratings displayed very strong positive correlation with the ground truth labels (Tau = 0.893 [0.653 – 1.000]), comparable to the correlation of both speech therapists’ ratings with the ground truth (Tau = 0.931 [0.745 – 1.000]; Tau = 0.881 [0.730 – 1.000]) and between themselves (Tau = 0.848 [0.653 – 1.000]) (Table 3). Whisper-ST1’s fully aligned with one of the speech therapists on the ratings of normal/mild and moderate dysarthria while tended to underscore the therapist’s rating of more severe cases (Fig 2). Similarly, Whisper-ST1’s fully aligned with the second speech therapist on the ratings of moderate and severe dysarthria while underscored the profound and overscored the normal/mild ratings of the therapist (Fig 2).

**Table 3:** Correlation and inter-rater metrics.

Pair	Sample level			Speaker level^#^		
	n	Kendall's Tau	Weighted Cohen’s Kappa	n	Kendall's Tau	Weighted Cohen’s Kappa
ST1 vs ST2	66	0.846	0.824	9	0.848 [0.653 – 1.000] / 99.99%	0.851 [0.550 – 1.000] / 100.0%
ST1 vs Whisper-ST1	66	0.689	0.701	9	0.822 [0.577 – 1.000] / 99.93%	0.814 [0.400 – 1.000] / 100.0%
ST1 vs Ground Truth	66	0.884	0.907	9	0.931 [0.745 – 1.000] / 100.0%	0.946 [0.727 – 1.000] / 100.0%
ST2 vs Whisper-ST1	66	0.734	0.734	9	0.878 [0.720 – 1.000] / 99.93%	0.878 [0.647 – 1.000] / 99.99%
ST2 vs Ground Truth	66	0.895	0.904	9	0.881 [0.730 – 1.000] / 99.99%	0.901 [0.690 – 1.000] / 100.0%
Whisper-ST1 vs Ground Truth	66	0.789	0.796	9	0.893 [0.653 – 1.000] / 99.93%	0.877 [0.571 – 1.000] / 100.0%

^**#**^**Per-speaker**: Point estimate [95% CI from 10,000 bootstrap samples] / % Valid bootstrap samples.

**Fig 1:**
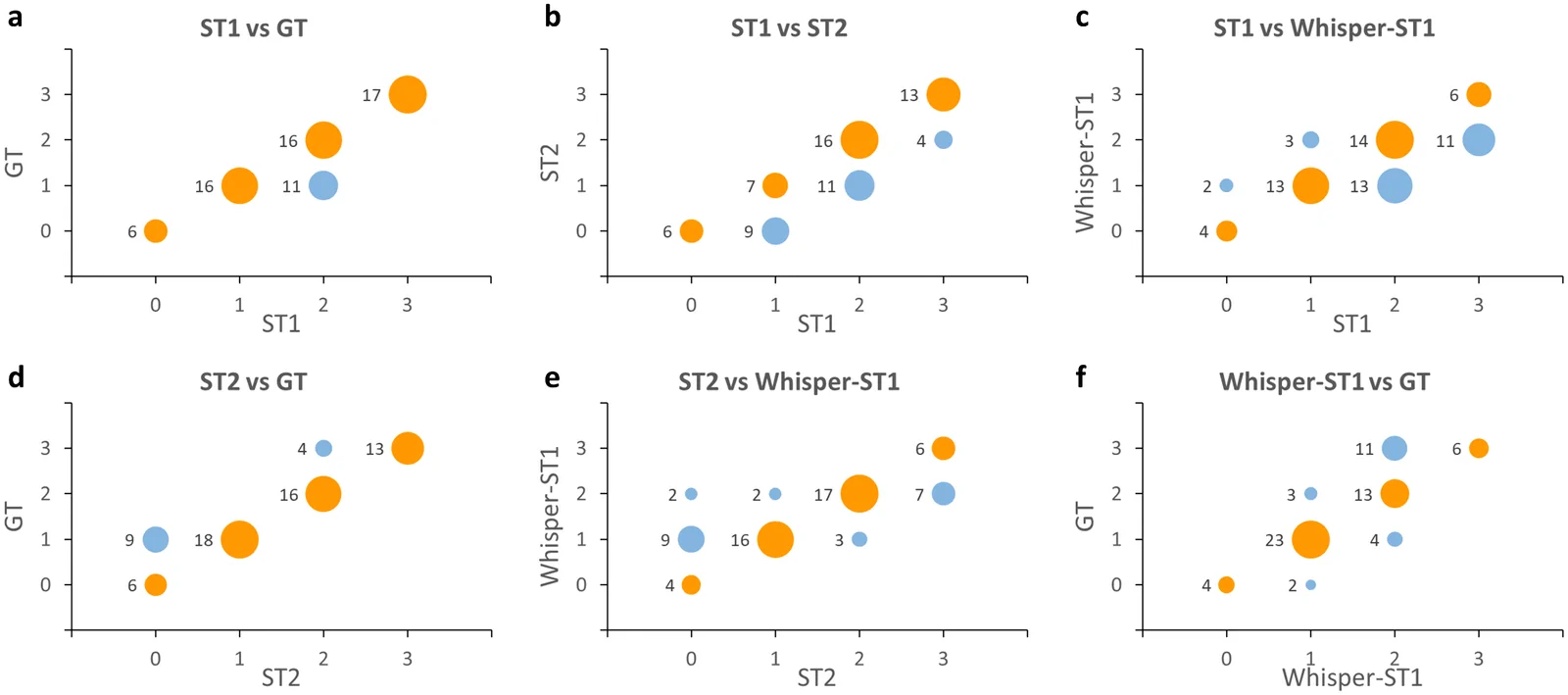
Sample-level correlation analysis between dysarthria severity raters. A total of 66 samples from the test set were independently rated by 2 speech therapists (ST1, ST2) and Whisper-ST1. Ground truth (GT) rating was assigned as described in Methods. Bubble plots were constructed to visualise the scoring correlations between raters. **a.** ST1 vs GT; **b.** ST1 vs ST2; **c.** ST1 vs Whisper-ST1; **d.** ST2 vs GT; **e.** ST2 vs Whisper-ST1; **f.** Whisper-ST1 vs GT. The position of the bubbles represents the dysarthria severity assigned by two independent raters (or by one rater and the ground truth), while the area of the bubbles and accompanying labels represent the counts of concordant (orange) and discordant (blue) ratings. 0: normal/mild; 1: moderate; 2: severe; 3: profound.

**Fig 2:**
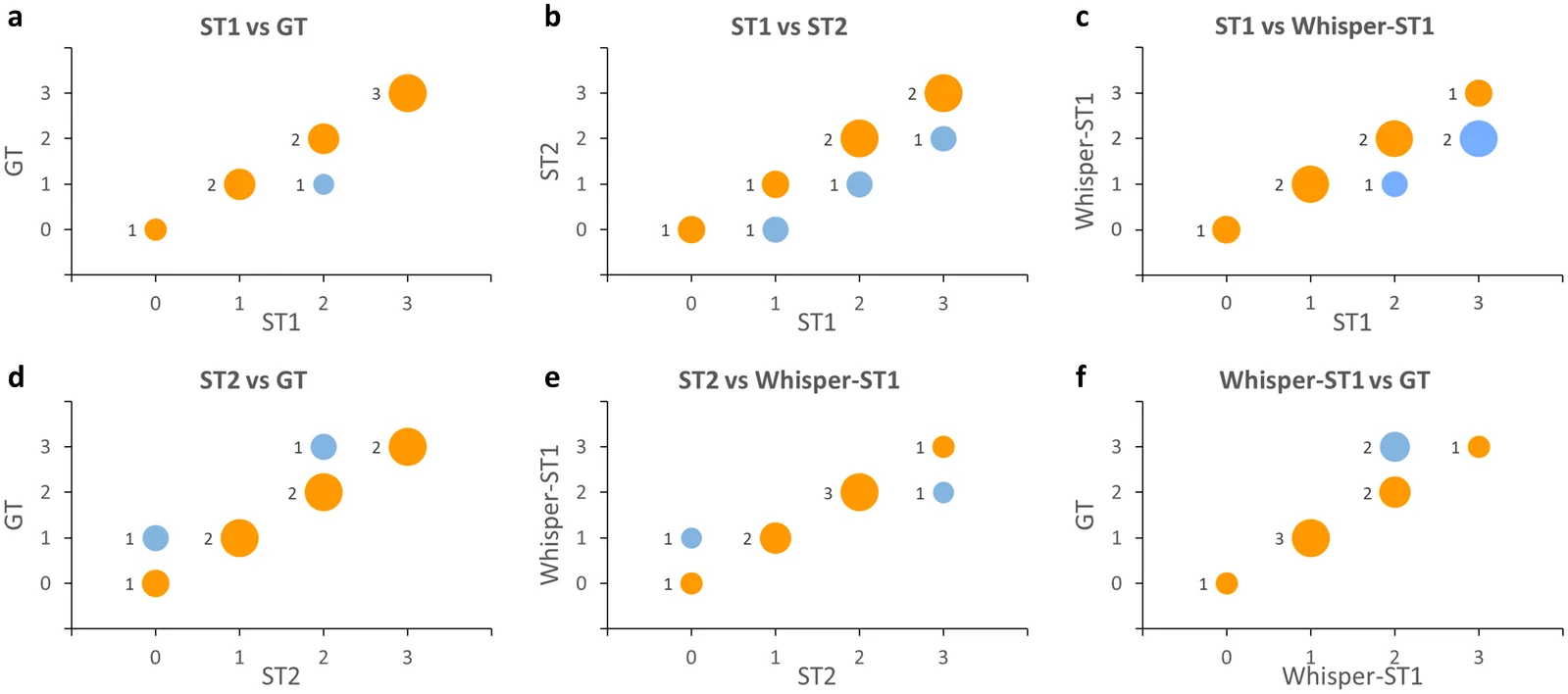
Speaker-level correlation analysis between dysarthria severity raters. A total of 9 speakers from the test set were independently rated by 2 speech therapists (ST1, ST2) and Whisper-ST1. Ground truth (GT) rating was assigned as described in Methods. Bubble plots were constructed to visualise the scoring correlations between raters. **a.** ST1 vs GT; **b.** ST1 vs ST2; **c.** ST1 vs Whisper-ST1; **d.** ST2 vs GT; **e.** ST2 vs Whisper-ST1; **f.** Whisper-ST1 vs GT. The position of the bubbles represents the dysarthria severity assigned by two independent raters (or by one rater and the ground truth), while the area of the bubbles and accompanying labels represent the counts of concordant (orange) and discordant (blue) ratings. 0: normal/mild; 1: moderate; 2: severe; 3: profound.

### Inter rater agreement

At sample level, Whisper-ST1 showed substantial agreement with both speech therapists in the rating of dysarthria (Weighted kappa = 0.701 and 0.734), although lower than the agreement between the two experts (Weighted kappa = 0.824) (Table 3). This mild discrepancy disappeared at the speaker level, where Whisper-ST1 showed agreements with both therapists (Weighted kappa = 0.814 [0.400 – 1.000] and 0.878 [0.647 – 1.000]) comparable to the inter-therapists’ agreement (Weighted kappa = 0.851 [0.550 – 1.000]), indicating that Whisper-ST1 is a reliable model for dysarthria severity classification, especially when rating multiple audio records from the same speaker.

### Ablation experiments

The methodology to develop Whisper-ST1 incorporates the following elements: 1) A rich and diverse speech dataset from social media and research sources; 2) A regression loss for ordinal classification; 3) A progressive fine-tuning strategy (Methods). To dissect the contributions of individual pipeline components to model performance, we conducted ablation experiments (Tables A-K in S1 Appendix). While excluding the TORGO data from the training set had a moderate effect on speaker-level performance (7% drop in F1, 0.113 in Tau, and 0.068 in Weighted kappa), removing the social media data had a substantial impact (39% drop in F1, 0.270 in Tau, and 0.377 in Weighted kappa). Likewise, reverting to conventional cross-entropy loss reduced performance markedly (16% drop in F1, 0.204 in Tau, and 0.142 in Weighted kappa). Notably, fine tuning the full model or the classification head alone decreased F1 score by 30% and 55%, Tau by 0.270 and 0.546, and Weighted kappa by 0.220 and 0.669, respectively. These finding indicate that all the components of the training pipeline are necessary to achieve superior model accuracy and agreement with human expertise. Therefore, Whisper-ST1 performance can be attributed to the combination of the methodological elements proposed in this study, with higher weightage to the fine-tuning strategy, followed by the contributions of the social media data, the regression loss, and, to a lesser extent, the TORGO data.

## Discussion

To address the rising need for speech therapy in the Singapore’s healthcare system [4,37], scalable adjustive digital AI solutions that can sustain service delivery are increasingly essential [38,39]. Speech disorders such as dysarthria require intensive, task-specific and repetitive practice to achieve meaningful clinical outcomes (10, 11). However, the delivery of recommended intensity in routine clinical settings remains constrained by therapy cost, resources availability, and patient adherence. This study describes the development and evaluation of Whisper-ST1, an AI model trained in classifying the severity of dysarthric speech. Whisper-ST1 showed substantial agreement with speech therapists in the rating of dysarthria severity, supporting the feasibility of the approach. Accuracy was higher for normal/mild and moderate cases and dropped moderately for severe and profound dysarthria. Misclassifications deviated by only one adjacent severity level rather than randomly, mainly underrating profound as severe, showing that the model preserves ordinal progression and captures severity gradients. Even in expert perceptual assessment, the differentiation between severe and profound dysarthria may be subjective and influenced by intelligibility context [2,40]. The findings from the study demonstrate the feasibility of leveraging machine learning techniques to differentiate severity levels of dysarthria based on acoustic and speech features.

Performance of classification techniques largely depends on task complexity, from vowels and single words through read-speech and finally spontaneous-speech (9). In contrast to previous studies that relied solely on read-speech samples from research datasets, such as TORGO [32] or UA-Speech [31], our work leveraged more challenging spontaneous-speech data from social media content. While datasets used for research purposes are typically collected under controlled conditions and manually curated to meet quality standards, they may not reflect all the nuances of real-world scenarios, thus hindering their potential applicability in clinical settings. In contrast, files available in social media sites are typically noisier and have not been subject to strict data quality controls. Therefore, these factors increased the spectrum of recording conditions represented in the training data. In this regard, unlike studies confined to a single controlled corpus of clinical data, our study combined controlled TORGO recordings with spontaneous social media recordings.

Audio files were annotated by independent subject experts based on an intelligibility score assigned by matching the transcript of the audio file against the corresponding ground truth and applying defined severity thresholds. Although similar scoring methods were used by [20,28] to classify the UA-Speech database into four impairment categories, our approach transcribed entire voice passages instead of isolated words, thus adding contextual information to the transcription process.

Our dataset suffered from two main limitations: 1) small size and 2) speaker and class imbalance, which could potentially lead to overfitting and bias, respectively. To address these limitations, other studies have used multi-task regularization techniques [28–30]. While this strategy effectively reduces overfitting to speaker-specific features, it does not address potential class imbalances in the training dataset, leading the model to preferentially predict majority classes, Instead, we opted for an automated data balancing technique that prevents any class or speaker from dominating and is scalable to any dataset irrespective of the size and class distribution.

While manually engineered features may not capture all the distinct patterns of dysarthric speech, learning discriminatory features from scratch may be challenging with small size datasets, potentially overfitting the model to uninformative signal. Likewise, embeddings extracted by models pretrained for diverse audio tasks may not necessarily contain adequate features for the target task. In this study, we took advantage of a transformer architecture pretrained with a massive audio corpus for an ASR task, and thus capable of extracting rich speech features, and adapted it to a related audio classification task by fine-tuning, thus mitigating small-sample limitations. In contrast to Kadirvelu et al., 2025, who fine-tuned wav2vect [41], we leveraged a model of the Whisper family as backbone [36]. Whisper was trained with 680k hours of labeled multilingual speech data collected from the web, while the version of wav2vec used by Kadirvelu and colleagues was trained with 960 hours of unlabeled English audio data from the LibriSpeech corpus (LS-960) [42]. Whisper was therefore selected as the backbone model due to the size and linguistic diversity of its pretraining dataset. Literature has shown that ASR systems have underperformed when applied to dysarthric speech due to mismatch with models trained on normative speech and acoustic variability [43,44]. By creating a model specifically for pathological speech classification, this study advances efforts to bridge the gap between ASR and clinically significant speech analytics.

Most classifiers rely on categorical cross-entropy to measure the distance between ground truth labels and their predicted probabilities. However, this approach treats all types of prediction errors equally, resulting in potentially suboptimal loss estimation and parameter update for ordinal categories. Kadirvelu et al., 2025 introduced an additional contrastive loss to disentangle speaker from dysarthria-specific embeddings. However, this function fails to capture the ordered relationships of the dysarthria severity levels. We implemented a regression-based loss function which penalizes errors proportionally to the distance between predictions and true values. Regression modeling for ordinal classification of severity level is a novel approach in dysarthria classification research, which is dominated by binary and unordered multi-class techniques [28,45].

Classical fine-tuning methods for classification tasks, such as the one reported by Kadirvelu et al., 2025, involve tweaking the model architecture by inserting a classification head and updating either 1) the full model parameters or 2) only the new layers during training. However, by rigidly pre-setting the number of trainable parameters, these strategies may potentially result in 1) catastrophic forgetting of pre-learned patterns if the fine-tuning dataset is small or 2) incomplete adaptation to the classification task due to insufficient number of trainable parameters. Notably, our approach was designed to reduce the risk of forgetting relevant preexisting knowledge and adapt flexibly to the new task by gradually incrementing the number of trainable layers.

This proof-of-concept study has several important limitations that should be noted. First, the study did not include an independent external or cross-corpus test set. The held-out test speakers were drawn from the same social media collection and processing pipeline used for model development. Therefore, robustness across different recording environments and generalizability to independent clinical populations could not be established. Second, Whisper-ST1 shows higher accuracy in the classification of normal/mild and moderate dysarthria, and a tendency to classify profound dysarthria as severe, although the small number of test speakers limits conclusions about performance within individual severity groups. The severe and profound categories contained relatively few test speakers, limiting the precision and increasing the uncertainty of performance estimates in these groups. These errors may have clinically important consequences, as they may lead to recommended exercises of excessive complexity to individuals in the highest severity tier above their speech competency, thus causing patient frustration [2,29]. Larger clinical cohorts are needed to assess performance across the full severity range. Third, although social media speech increased the range of recording conditions, our dataset includes mainly standard English and does not represent the diversity of accents that pervade multilingual and multiethnic nations such as Singapore, characterized by a unique tone, pronunciation, and rhythm. Those traits of the spoken language may confound the AI model, which may fail to discriminate class features from accent features, leading to incorrect severity rating. Likewise, our model was only trained and tested with English data. Generalizability to other languages remains unknown. Fourth, Whisper-ST1 was not trained to recognize and reject non-speech audio files. Augmenting the dataset with non-speech samples or stacking an ensemble of models to handle separate tasks could potentially facilitate the integration of Whisper-ST1 into real-world clinical workflows. Fifth, it is unknown whether Whisper-ST1 is sensitive enough to detect within-patient changes over time, which could be assessed by the fraction of samples rated within each severity level. If our model can successfully detect measurable improvement after a given therapy period, the adaptive difficulty of the exercises derived from this assessment may be more relevant clinically. Future work should validate longitudinal sensitivity and user adherence. Sith, audio data gives only a partial overview of the overall speech competency. Future refinements could also incorporate other data modalities, including video of lip movements or respiratory data via wearable sensors to provide a more holistic assessment of motor speech subsystems including respiration, phonation, and articulation. Seventh, since the main demographic segment of dysarthria rehabilitation is aged 65 and above, future research needs to investigate the aspect of human-AI interaction to ascertain how elderly react to machine-generated ratings and recommendations. The psychological impact of AI feedback should also be an area of active exploration. Lastly, this study assessed technical performance rather than clinical effectiveness. The work did not evaluate real-world deployment, longitudinal sensitivity to change, or user interaction, all of which are critical for clinical translation. As such, the current findings should be interpreted as preliminary and hypothesis-generating rather than definitive evidence of clinical readiness. Prospective validation is required to determine potential safety, usability, and effectiveness in clinical or home-based rehabilitation.

Whisper-ST1 has the potential to be incorporated into a speech therapy mobile application to support remote speech practice at home for patients with dysarthria. However, the present study did not assess features that are required to develop a speech rehabilitation tool, such as real-time feedback, personalized exercise recommendation, longitudinal monitoring, or clinical effectiveness. Likewise, the agreement with expert raters warrants further evaluation of Whisper-ST1 for adaptive therapy and objective monitoring. Future studies should also examine whether repeated recordings improve classification performance. Importantly, this AI model is not intended to replace clinical expertise but to augment it. Responsible deployment of AI in healthcare emphasizes the use of AI in decision support rather than complete automation of judgment [38]. Prospective studies are thus needed to determine whether the automation of severity assessment and consistent scoring can support home monitoring alongside clinician assessment.

To address some of the current limitations of Whisper-ST1 additional studies have been designed to re-train the model with a larger sample of local normative data across dialect groups, including diverse English accents prevalent in Singapore, and test its effectiveness in clinical workflows. Future prospective studies should evaluate the model in larger independent clinical cohorts, including greater representation of severe and profound dysarthria. Home-based studies are also needed to determine whether the model can detect changes over time and whether its assessments can be used safely and effectively during rehabilitation. If clinically validated, automated dysarthria severity assessment may support accessibility and continuity of speech therapy rehabilitation services for patients with dysarthria.

## Conclusion

This proof-of-concept study demonstrated that Whisper-ST1, an ASR model adapted for dysarthria severity classification through transfer learning, shows substantial agreement with expert speech therapists (Weighted Kappa = 0.814 [0.400 – 1.000] and 0.878 [0.647 – 1.000]), comparable to inter-therapist agreement (Weighted Kappa = 0.851 [0.550 – 1.000]). The combination of research and real-world data increased the range of speech and recording conditions represented during model development, and improved overall performance, as confirmed by ablation experiments. These findings hold promise for the development of an AI assisted speech rehabilitation and monitoring tool. However, the present study did not establish robustness across acoustic environments, generalizability to external populations, or clinical effectiveness. Prospective validation in independent, larger, and more linguistically diverse cohorts will be required to assess the clinical value of Whisper-ST1.

## Materials and methods

### Data collection

The dataset used in this study was collected from two main public sources (Table 4):

**Table 4:** Dataset description.

Dataset	Condition	Number of speakers	Number of speaker-sessions	Total number of samples	Number of speakers^1^ selected	Number of samples after selection	Number of samples after partition
TORGO	Dysarthria	8	15	3321	12 total 3 mild 2 moderate 3 severe 4 profound	600 total 196 mild 139 moderate 123 severe 142 profound	194 total 24 mild 36 moderate 57 severe 77 profound
	Control	7	15	5862	0 normal	0 normal	0 normal
	**Total**	**15**	**30**	**9183**	**12**	**600**	**194**
SM	Dysarthria	76	–	1953 total 685 mild 587 moderate 442 severe 239 profound	62 total 14 mild 20 moderate 16 severe 12 profound	850 total 306 mild 293 moderate 126 severe 125 profound	508 total 101 mild 210 moderate 110 severe 87 profound
	Control	26	–	416 normal	24 normal	213 normal	121 normal
	**Total**	**102**	**–**	**2369**	**86**	**1063**	**629**
**Total**		**117**	**30**	**11552**	**98**	**1663**	**823**

^1^In the TORGO dataset, speakers refer to individual speaker-session pairs.

Social Media (SM): For the purpose of this study, a total of 89 publicly available recordings of dysarthric speech were identified and collected from social media platforms, including YouTube (https://www.youtube.com), TikTok (https://www.tiktok.com), and Instagram (https://www.instagram.com) and used according to their Terms and Conditions. The recordings comprise either monologues or dialogues of up to 8 speakers spanning from 0.5 to 24 minutes.

TORGO: Public dataset comprising 9183 audio samples from 8 speakers with either cerebral palsy or amyotrophic lateral sclerosis and 7 controls. Audio files were recorded throughout a period of 3 years in either single or multiple sessions, totalling 30 speaker-session pairs [32]. The dataset includes audio files with non-words, short words, restricted sentences, and unrestricted sentences, spanning from 1 to 43 seconds. Each sample is matched to a textual prompt containing the reference transcript of the audio file.

Although the majority of files have an audio frequency of 16,000 Hz, some samples were recorded at different rates. To standardise the frequency for training and inference, the feature extractor of Whisper-ST1 resamples the input data to 16,000 Hz (see Model selection and fine-tuning)

### Ratings of speech samples

Each speech sample was assigned to a dysarthria severity rating (normal, mild, moderate, severe, or profound). Speech intelligibility, defined as how clearly the listener can comprehend what the speaker is saying [46] is often used as an indicator of severity of speech disorder (i.e., lower speech intelligibility score translates to more severe dysarthria). One widely acceptable measurement of intelligibility is orthographic transcription, which involves counting the number of correctly perceived words by the listener, ideally a speech therapist [47].

A total of 8 speech therapists with at least 1 year of experience treating patients with dysarthria were recruited from an acute hospital and rehabilitation facility in Singapore for sample labeling. Two speech therapists were assigned randomly to one labelling group, with a total of 4 groups. To reduce the impact of listener fatigue, each ST pair was assigned to listen and rate a maximum of 30 speech samples. During each labelling session, the audio clips were played from the same device, and volume was set to the maximum. Raters had no access to reference transcripts. Each sample (1–5 sentences) was played once.

For the SM dataset, the reference transcript was completed by 2 speech therapists in the research team, through various rounds of iteration to ensure that the transcript is as accurate as possible. For the TORGO dataset, the reference transcript was directly extracted from the respective audio prompts. The number of correctly perceived words was matched against the total number of words in the reference transcript and the percentage of correctly perceived words by each speech therapist was computed for each sample. An average was then obtained across the two speech therapists to assign the final percentage intelligibility. The percentage intelligibility was used to derive the dysarthric severity rating for each sample using a thresholding criterion (Table 5). The classification criterion was adapted from a prior study by Stipancic and colleagues [48], who proposed specific ranges of percentage intelligibilities for each severity rating (normal, mild, moderate, severe, profound). In the present study, mild and normal ratings were condensed into one group, termed “normal/mild”. This was done in view of two considerations: 1) To better reflect actual clinical practice in assigning severity scores, where “mild” dysarthria often retains high intelligibility, making it acoustically similar to “normal” speech; 2) To reduce the number of class labels for model training due to the relatively small number of samples gathered from publicly available sources.

**Table 5:** Classification of dysarthria severity based on percentage speech intelligibility.

Percentage intelligibility	Proposed severity rating
85% - 100%	normal /mild
70% - 84%	moderate
45% - 69%	severe
0% - 44%	profound

For the SM dataset, one severity rating was assigned per speaker, while for the TORGO dataset rating was assigned per speaker-session pair, as dysarthria severity might evolve during the intervals between sessions. For the SM dataset, the full recording of each speaker was selected to assign intelligibility, whereas for the TORGO dataset five samples of restricted sentences from each speaker-session pair were selected to compute intelligibility. Ground truth dysarthria severity was assigned per speaker and speaker-session as the rating of the average intelligibility scored by both therapists across the selected samples from the speaker or speaker-session. Subsequently, all the individual audio files extracted from the SM and the TORGO datasets (see next section “Data preprocessing”) were assigned the rating from their respective speaker or speaker-session, respectively. To ensure clarity, both “per speaker” and “per speaker-session” analyses are referred to as “speaker-level” when reporting AI model performance.

### Data preprocessing

Recordings from the SM dataset were diarized and segmented using Sortformer Diarizer 4spk v1 [49] to extract audio samples containing speech from a single speaker and to exclude silence periods. The diarized audio segments were manually curated to remove files containing more than one speaker voice or no recognizable voice. Upon segmentation, a total of 1953 audio segments from 62 dysarthric speakers and 416 segments from 24 control individuals were extracted (Table 4). Subsequently, two filters were applied to select audio files for model development:

1. From each recording, only one dysarthric speaker and one or no control speakers were selected to prevent overrepresentation of samples from any particular recording containing multiple speakers in the dataset.
2. From each speaker, only audio segments with a duration of at least 2 seconds were selected to ensure sufficient number of acoustic features in the sample for model training. Only three SM segments in the final dataset did not meet the length criterion but were included to increase speaker representation from the SM data. Subsequently, the fidelity of individual selected segments to their original speaker severity rating was manually verified

All segments belonging to the same speaker were assigned identical dysarthria severity label (see previous section “Ratings of speech samples”). The final diarized and filtered SM dataset comprised 1063 audio segments (125 profound, 126 severe, 293 moderate, 306 mild, and 213 control) from 86 speakers.

Audio samples from the TORGO dataset were used without further processing as they only contain one speaker and no significant silence periods. Two filters were applied to select audio files for model development:

1. Out of the 30 speaker-session pairs, 12 with dysarthria rating were selected to retain a balanced distribution of severity levels.
2. From each speaker-session pair, only audio files meeting the following criteria were selected:

- The corresponding text prompt file is available and in a txt format.
- The prompt is not a bracketed annotation (e.g., “[laughing]”).
- The cleaned prompt (after excluding alphanumeric characters) contains more than 10 characters.
- The audio file duration is > 0.5 sec and < 40 sec.

All segments belonging to the same speaker-session were assigned identical dysarthria severity label (see previous section “Ratings of speech samples”). The final TORGO dataset comprised 600 audio segments (142 profound, 123 severe, 139 moderate, 196 mild) from 12 speaker-sessions.

### Data partition

Due to the small size and class imbalance of the two dysarthria datasets, a partition strategy was designed to preserve a balance between the representation of the different severity levels and speakers in the train, validation, and test sets, including the following considerations:

- Universal patient preservation: All speakers are represented in the dataset without any drop.
- Speaker sample capping: Applies a soft cap (20 samples) to prevent individual speakers from dominating.
- Patient-level splitting: Each speaker is assigned to only one split to avoid information leakage between datasets.
- Constraint enforcement: Ensures that each class contains at least one speaker in each split.
- Class balance protection and dominance capping: Limits class size to 135% of the average class size to prevent overrepresentation of any severity level.
- Split ratio enforcement: Maintains an approximate 80:10:10 ration between train, validation, and test sets.

Adhering to these considerations, the selected samples from the two dysarthria datasets (Table 4) were split into the final train. validation, and test sets (Table 6). Overall, the development set contained 629 audio samples (87 profound, 110 severe, 210 moderate, and 222 normal/mild) from 86 speakers of the SM dataset and 194 samples (77 profound, 57 severe, 36 moderate, and 24 normal/mild) from 12 speaker-sessions of the TORGO dataset (Table 6).

**Table 6:** Dataset partition.

Dataset	Class	Train		Valid		Test		Total	
		Samples	Speakers^1^	Samples	Speakers^1^	Samples	Speakers^1^	Samples	Speakers^1^
TORGO	Norm/Mild	24	3	0	0	0	0	24	3
	Moderate	36	2	0	0	0	0	36	2
	Severe	57	3	0	0	0	0	57	3
	Profound	77	4	0	0	0	0	77	4
	**Total**	**194**	**12**	**0**	**0**	**0**	**0**	**194**	**12**
SM	Norm/Mild	175	30	24	4	23	4	222	38
	Moderate	161	15	22	2	27	3	210	20
	Severe	78	12	16	2	16	2	110	16
	Profound	53	7	17	2	17	3	87	12
	**Total**	**467**	**64**	**79**	**10**	**83**	**12**	**629**	**86**
**Total**	**Total**	**661**	**76**	**79**	**10**	**83**	**12**	**823**	**98**

^1^In the TORGO dataset, speakers refer to individual speaker-session pairs.

### Model selection and fine-tuning

Two main considerations guided the selection of the model architecture and the training strategy:

1) Given the rich set of features embedded in speech data, a deep learning architecture was preferred, as neural networks can learn complex hidden features from audio samples that may not be apparent or simple to engineer;
2) Due to the relatively small size of the training dataset, a transfer learning approach was selected, where a model pre-trained with a large and diverse corpus for a particular task is fine-tuned with a small domain-specific dataset to a different but related task, instead of training a naïve model from scratch [50]. This technique takes advantage of the pre-trained model parameters, which have learned relevant patterns from the original task data, and updates them to optimise performance on the new task, thus reducing the time and compute power required to train the model.

Following these principles, we selected Whisper, a family of models for ASR and speech translation with a backbone transformer architecture [36]. Whisper was trained on 680k hours of labeled speech data using large-scale multilingual and multitask weak supervision [36]. Whisper models comprise a feature extractor and an encoder-decoder for sequence-to-sequence tasks. The feature extractor resamples the input audio files to 16,000 Hz and converts the signal data into an 80-channel mel spectrogram, which is subsequently processed by a shallow convolutional neural network to extract the features that are passed to the transformer encoder blocks. Whisper comprises a suite of models of various sizes ranging from 39M to 1550M parameters. Prompted by the small size of our training dataset and to minimize potential overfitting, the base architecture of the Whisper model family was selected for this study, comprising 6 encoder-decoder layers with a width of 512 and 8 attention heads each, totalling 74M parameters. We adapted the Whisper model architecture to a classification task by inserting a classification head after the linear projection layer.

Whisper-base was fine-tuned using the labeled training and validation datasets. The training methodology included the following techniques:

1. Gradually unfreezing the classification and projector heads plus an increasing number of preceding layers.
2. As the class labels are ordinally arranged (from lower to higher severity) and the magnitude of misclassification errors is important, a regression loss function (Mean Squared Error - MSE) was selected instead of the traditional categorical cross-entropy used to train classification models. This function penalises proportionally to the distance between the predicted severity level and the ground truth. In this regression approach, class labels are mapped to ordinal labels (“normal/mild”: 0; “moderate”: 1; “severe”: 2; “profound”: 3) used to train the algorithm. During training, output logits are matched to the ground truth label to compute the MSE and backpropagate the errors to update the model parameters. During inference, output logits are rounded to the nearest integer and re-mapped to the original class labels to obtain the model predictions.

Multiple rounds of fine-tuning were conducted to optimise the training hyperparameters. Upon optimisation, Whisper-ST1 was developed in three rounds of fine-tuning by gradually unfreezing 4, 6, and 8 hidden layers, using the following set of hyperparameters: Epochs: 30; Early stopping: 8; Learning rate: 5e-05; Warm-up ratio: 0.1; Batch size 16; Gradient accumulation steps: 2; Weight decay: 0.001. The maximum length of the input audio files was set to 30 seconds.

### Performance evaluation

#### Classification metrics.

Model performance across multiple metrics was assessed at sample and at speaker level using the 83 audio samples from 12 speakers in the test set. At sample level, individual sample predictions were matched against their respective ground truth. At speaker level, predictions were determined by the majority label predicted across all samples of the speaker and matched against the speaker ground truth. When there was a tie in the majority vote, the speaker rating was decided by selecting the nearest severity level to the average rating of all the speaker samples.

Performance was reported using standard metrics for multiclass classification tasks, including overall Accuracy as well as Precision, Recall, and F1 score (the harmonic mean of precision and recall), which is less biased than Accuracy by class imbalance, for each class, and overall (weighted and macro-average).

#### Correlation analyses.

The correlation between the dysarthria ratings from Whisper-ST1 and two Speech Therapists was assessed by computing Kendall’s Tau at sample and speaker granularity levels using 66 samples from 9 speakers from the test set (17 samples from 3 control speakers were excluded from this analysis as they had not been formally rated by the Speech Therapists). Controls were automatically assigned to the “normal/mild” severity group. Therefore, correlation and inter-rater reliability metrics were not computed for these samples. In contrast, standard classification metrics were computed for Controls, as these metrics only require a single ground truth label. Kendall’s Tau is preferred over Pearson’s r for measuring monotonic relationships between ordinal variables, such as dysarthria severity levels, instead of continuous variables, and over Spearman’s Rho for small datasets and when many ties exist.

#### Inter rater reliability analyses.

The level of agreement in dysarthria severity classification between Whisper-ST1 and two Speech Therapists was assessed by computing weighted Cohen’s kappa at sample and speaker granularity levels using 66 samples from 9 speakers from the test set. Weighted Cohen’s kappa is preferred over standard Cohen’s kappa for ordinal data as it applies weights to penalise larger disagreements more severely and vice-versa. Weighted Cohen’s kappa was computed applying quadratic weight penalties.

#### Speaker level CIs.

Speaker level bootstrapping was performed to compute 95% CIs of the classification, correlation, and agreement metrics. A total of 10,000 bootstrap samples were generated by resampling with replacement from the test set cohorts of 12 speakers (classification metrics) and 9 speakers (correlation and agreement metrics). To compute per-class classification metrics (Precision, Recall, F1 score) from each sample, the following convention was used to account for special scenarios derived from the randomness of the resampling method: 1) For samples without true instances of one or more class labels, Precision was assigned a value of 0, while Recall and F1 score were left undetermined for those classes; 2) For samples without predicted instances of one or more class labels, Precision, Recall, and F1 score were assigned a value of 0; 3) For samples without both true and predicted instances of one or more class labels, Precision, Recall, and F1 score were left undetermined for those classes. Macro-average metrics per sample were computed by averaging only over the classes actually determined in the sample. Accuracy and weighted-average metrics per sample were determined for all scenarios. To compute correlation and agreement metrics (Kendall’s Tau and Weighted Cohen’s kappa) from each sample, the following convention was used to account for special scenarios derived from the randomness of the resampling method: 1) For samples where one rater assigned a single class label to all instances and the second rater assigned either multiple classes or a single, but different, class label to all instances Kendall’s Tau was left undetermined, while Weighted Cohen’s kappa was assigned a value of 0; 2) For samples where both raters assigned the same single class label to all the instances, both metrics were left undetermined. Performance metrics were computed from each bootstrap replicate and the 2.5th and 97.5th percentiles were extracted over the replicates where the metric was determined to obtain the lower and upper 95% CIs, respectively. The percent of valid replicates for each metric was also reported.

### Ablation experiments

The individual contributions of the fine-tuning pipeline components to overall model performance were assessed in isolation by a series of ablation experiment. Single components of the training pipeline were removed or replaced by conventional approaches, while fixing the remaining components, as follows:

- Ablation 1: Replacement of the regression loss by categorical cross-entropy loss.
- Ablation 2: Exclusion of the TORGO samples from the training dataset.
- Ablation 3: Exclusion of the social media samples from the training dataset.
- Ablation 4: Fine-tuning all the model layers.
- Ablation 5: Fine-tuning only the classification head and freezing the Whisper-base backbone as a feature extractor

Subsequently, the same base model (Whisper-base) was fine-tuned under the ablated conditions and evaluated as described in the previous section using exactly the same test set.

## Supporting information

S1 Appendix Appendix Table A–Table K. **Table A:** Whisper-ST1 (Ablation 1) performance metrics. **Table B:** Whisper-ST1 (Ablation 1) confusion matrices. **Table C:** Whisper-ST1 (Ablation 2) performance metrics. **Table D:** Whisper-ST1 (Ablation 2) confusion matrices. **Table E:** Whisper-ST1 (Ablation 3) performance metrics. **Table F:** Whisper-ST1 (Ablation 3) confusion matrices. **Table G:** Whisper-ST1 (Ablation 4) performance metrics. **Table H:** Whisper-ST1 (Ablation 4) confusion matrices. **Table I:** Whisper-ST1 (Ablation 5) performance metrics. **Table J:** Whisper-ST1 (Ablation 5) confusion matrices. **Table K:** Comparison of full and ablated versions of Whisper-ST1 correlation and inter-rater metrics.
(DOCX)
