# Supplementary material for: An artificial intelligence-based audio recognition system for dysarthria home-based rehabilitation: A proof-of-concept study
Source: PLOS Digit Health. 2026 Oct 21;5(10):e0001794. doi: 10.1371/journal.pdig.0001794 (PMC13686279; doi:10.1371/journal.pdig.0001794)
Supplement: Supplementary file 1 [file pdig.0001794.s001.docx]

**Table A: Whisper-ST1 (Ablation 1) performance metrics**

|  |  | **Sample level*** | | | |  | **Speaker level*** | | | | |  |
| --- | --- | --- | --- | --- | --- | --- | --- | --- | --- | --- | --- | --- |
|  |  | | **Precision** | **Recall** | **F1** | | |  | **Precision** | **Recall** | **F1** | |
| Norm/Mild |  | | 0.91 | 0.91 | 0.91 | | |  | 1.00 | 1.00 | 1.00 | |
| Moderate |  | | 0.71 | 0.37 | 0.49 | | |  | 1.00 | 0.33 | 0.50 | |
| Severe |  | | 0.38 | 1.00 | 0.55 | | |  | 0.33 | 1.00 | 0.50 | |
| Profound |  | | 1.00 | 0.24 | 0.38 | | |  | 1.00 | 0.33 | 0.50 | |
| **Macro Average** |  | | **0.75** | **0.63** | **0.58** | | |  | **0.83** | **0.67** | **0.63** | |
| **Weighted Average** |  | | **0.76** | **0.61** | **0.60** | | |  | **0.89** | **0.67** | **0.67** | |

***Overall accuracy**: Per-sample: 61%; Per-speaker: 67%

**Table B: Whisper-ST1 (Ablation 1) confusion matrices**

|  |  | **Sample level** | | | |  | **Speaker level** | | | |
| --- | --- | --- | --- | --- | --- | --- | --- | --- | --- | --- |
|  |  | Predicted | | | |  | Predicted | | | |
|  |  | Norm/Mild | Moderate | Severe | Profound |  | Norm/Mild | Moderate | Severe | Profound |
| Actual | Norm/Mild | 21 | 2 | 0 | 0 |  | 4 | 0 | 0 | 0 |
|  | Moderate | 1 | 10 | 16 | 0 |  | 0 | 1 | 2 | 0 |
|  | Severe | 0 | 0 | 16 | 0 |  | 0 | 0 | 2 | 0 |
|  | Profound | 1 | 2 | 10 | 4 |  | 0 | 0 | 2 | 1 |

**Table C: Whisper-ST1 (Ablation 2) performance metrics**

|  |  | **Sample level*** | | | |  | **Speaker level*** | | | | |  |
| --- | --- | --- | --- | --- | --- | --- | --- | --- | --- | --- | --- | --- |
|  |  | | **Precision** | **Recall** | **F1** | | |  | **Precision** | **Recall** | **F1** | |
| Norm/Mild |  | | 0.91 | 0.87 | 0.89 | | |  | 1.00 | 1.00 | 1.00 | |
| Moderate |  | | 0.70 | 0.52 | 0.60 | | |  | 1.00 | 0.67 | 0.80 | |
| Severe |  | | 0.43 | 0.94 | 0.59 | | |  | 0.4 | 1.00 | 0.57 | |
| Profound |  | | 1.00 | 0.35 | 0.52 | | |  | 1.00 | 0.33 | 0.50 | |
| **Macro Average** |  | | **0.76** | **0.67** | **0.65** | | |  | **0.85** | **0.75** | **0.72** | |
| **Weighted Average** |  | | **0.77** | **0.66** | **0.66** | | |  | **0.90** | **0.75** | **0.75** | |

***Overall accuracy**: Per-sample: 66%; Per-speaker: 75%

**Table D: Whisper-ST1 (Ablation 2) confusion matrices**

|  |  | **Sample level** | | | |  | **Speaker level** | | | |
| --- | --- | --- | --- | --- | --- | --- | --- | --- | --- | --- |
|  |  | Predicted | | | |  | Predicted | | | |
|  |  | Norm/Mild | Moderate | Severe | Profound |  | Norm/Mild | Moderate | Severe | Profound |
| Actual | Norm/Mild | 20 | 3 | 0 | 0 |  | 4 | 0 | 0 | 0 |
|  | Moderate | 2 | 14 | 11 | 0 |  | 0 | 2 | 1 | 0 |
|  | Severe | 0 | 1 | 15 | 0 |  | 0 | 0 | 2 | 0 |
|  | Profound | 1 | 2 | 9 | 6 |  | 0 | 0 | 2 | 1 |

**Table E: Whisper-ST1 (Ablation 3) performance metrics**

|  |  | **Sample level*** | | | |  | **Speaker level*** | | | | |  |
| --- | --- | --- | --- | --- | --- | --- | --- | --- | --- | --- | --- | --- |
|  |  | | **Precision** | **Recall** | **F1** | | |  | **Precision** | **Recall** | **F1** | |
| Norm/Mild |  | | 1.00 | 0.43 | 0.61 | | |  | 1.00 | 0.50 | 0.67 | |
| Moderate |  | | 0.20 | 0.15 | 0.17 | | |  | 0.00 | 0.00 | 0.00 | |
| Severe |  | | 0.30 | 0.81 | 0.43 | | |  | 0.29 | 1.00 | 0.44 | |
| Profound |  | | 0.78 | 0.41 | 0.54 | | |  | 1.00 | 0.33 | 0.50 | |
| **Macro Average** |  | | **0.57** | **0.45** | **0.44** | | |  | **0.57** | **0.46** | **0.40** | |
| **Weighted Average** |  | | **0.56** | **0.41** | **0.42** | | |  | **0.63** | **0.42** | **0.42** | |

***Overall accuracy**: Per-sample: 41%; Per-speaker: 42%

**Table F: Whisper-ST1 (Ablation 3) confusion matrices**

|  |  | **Sample level** | | | |  | **Speaker level** | | | |
| --- | --- | --- | --- | --- | --- | --- | --- | --- | --- | --- |
|  |  | Predicted | | | |  | Predicted | | | |
|  |  | Norm/Mild | Moderate | Severe | Profound |  | Norm/Mild | Moderate | Severe | Profound |
| Actual | Norm/Mild | 10 | 13 | 0 | 0 |  | 2 | 2 | 0 | 0 |
|  | Moderate | 0 | 4 | 21 | 2 |  | 0 | 0 | 3 | 0 |
|  | Severe | 0 | 3 | 13 | 0 |  | 0 | 0 | 2 | 0 |
|  | Profound | 0 | 0 | 10 | 7 |  | 0 | 0 | 2 | 1 |

**Table G: Whisper-ST1 (Ablation 4) performance metrics**

|  |  | **Sample level*** | | | |  | **Speaker level*** | | | | |  |
| --- | --- | --- | --- | --- | --- | --- | --- | --- | --- | --- | --- | --- |
|  |  | | **Precision** | **Recall** | **F1** | | |  | **Precision** | **Recall** | **F1** | |
| Norm/Mild |  | | 1.00 | 0.74 | 0.85 | | |  | 1.00 | 1.00 | 1.00 | |
| Moderate |  | | 0.44 | 0.26 | 0.33 | | |  | 0.00 | 0.00 | 0.00 | |
| Severe |  | | 0.36 | 1.00 | 0.53 | | |  | 0.29 | 1.00 | 0.44 | |
| Profound |  | | 1.00 | 0.35 | 0.52 | | |  | 1.00 | 0.33 | 0.50 | |
| **Macro Average** |  | | **0.70** | **0.59** | **0.56** | | |  | **0.57** | **0.58** | **0.49** | |
| **Weighted Average** |  | | **0.69** | **0.55** | **0.55** | | |  | **0.63** | **0.58** | **0.53** | |

***Overall accuracy**: Per-sample: 55%; Per-speaker: 58%

**Table H: Whisper-ST1 (Ablation 4) confusion matrices**

|  |  | **Sample level** | | | |  | **Speaker level** | | | |
| --- | --- | --- | --- | --- | --- | --- | --- | --- | --- | --- |
|  |  | Predicted | | | |  | Predicted | | | |
|  |  | Norm/Mild | Moderate | Severe | Profound |  | Norm/Mild | Moderate | Severe | Profound |
| Actual | Norm/Mild | 17 | 6 | 0 | 0 |  | 4 | 0 | 0 | 0 |
|  | Moderate | 0 | 7 | 20 | 0 |  | 0 | 0 | 3 | 0 |
|  | Severe | 0 | 0 | 16 | 0 |  | 0 | 0 | 2 | 0 |
|  | Profound | 1 | 3 | 8 | 6 |  | 0 | 0 | 2 | 1 |

**Table I: Whisper-ST1 (Ablation 5) performance metrics**

|  |  | **Sample level*** | | | |  | **Speaker level*** | | | | |  |
| --- | --- | --- | --- | --- | --- | --- | --- | --- | --- | --- | --- | --- |
|  |  | | **Precision** | **Recall** | **F1** | | |  | **Precision** | **Recall** | **F1** | |
| Norm/Mild |  | | 1.00 | 0.13 | 0.23 | | |  | 0.00 | 0.00 | 0.00 | |
| Moderate |  | | 0.37 | 0.93 | 0.53 | | |  | 0.30 | 1.00 | 0.46 | |
| Severe |  | | 0.23 | 0.19 | 0.21 | | |  | 0.50 | 0.50 | 0.50 | |
| Profound |  | | 0.00 | 0.00 | 0.00 | | |  | 0.00 | 0.00 | 0.00 | |
| **Macro Average** |  | | **0.40** | **0.31** | **0.24** | | |  | **0.20** | **0.38** | **0.24** | |
| **Weighted Average** |  | | **0.44** | **0.37** | **0.28** | | |  | **0.16** | **0.33** | **0.20** | |

***Overall accuracy**: Per-sample: 37%; Per-speaker: 33%

**Table J: Whisper-ST1 (Ablation 5) confusion matrices**

|  |  | **Sample level** | | | |  | **Speaker level** | | | |
| --- | --- | --- | --- | --- | --- | --- | --- | --- | --- | --- |
|  |  | Predicted | | | |  | Predicted | | | |
|  |  | Norm/Mild | Moderate | Severe | Profound |  | Norm/Mild | Moderate | Severe | Profound |
| Actual | Norm/Mild | 3 | 20 | 0 | 0 |  | 0 | 4 | 0 | 0 |
|  | Moderate | 0 | 25 | 2 | 0 |  | 0 | 3 | 0 | 0 |
|  | Severe | 0 | 13 | 3 | 0 |  | 0 | 1 | 1 | 0 |
|  | Profound | 1 | 9 | 8 | 0 |  | 0 | 2 | 1 | 0 |

**Table K: Comparison of full and ablated versions of Whisper-ST1 correlation and inter-rater metrics**

|  |  | **Sample level** | | |  | **Speaker level** | | | | | |  |
| --- | --- | --- | --- | --- | --- | --- | --- | --- | --- | --- | --- | --- |
| **Pair** |  | **n** | **Kendall's Tau** | **Weighted Cohen’s Kappa** |  | **n** | | **Kendall's Tau** | | **Weighted Cohen’s Kappa** | |  |
| ST1 vs ST2 |  | 66 | 0.846 | 0.824 |  | | 9 | | 0.848 | | 0.851 | |
| ST1 vs Ground Truth |  | 66 | 0.884 | 0.907 |  | | 9 | | 0.931 | | 0.946 | |
| ST2 vs Ground Truth |  | 66 | 0.895 | 0.904 |  | | 9 | | 0.881 | | 0.901 | |
| ST1 vs Whisper-ST1 (Full) |  | 66 | 0.689 | 0.701 |  | | 9 | | 0.822 | | 0.814 | |
| ST1 vs Whisper-ST1 (Ablation 1) |  | 66 | 0.438 | 0.509 |  | | 9 | | 0.729 | | 0.794 | |
| ST1 vs Whisper-ST1 (Ablation 2) |  | 66 | 0.531 | 0.611 |  | | 9 | | 0.669 | | 0.739 | |
| ST1 vs Whisper-ST1 (Ablation 3) |  | 66 | 0.483 | 0.498 |  | | 9 | | 0.623 | | 0.546 | |
| ST1 vs Whisper-ST1 (Ablation 4) |  | 66 | 0.460 | 0.546 |  | | 9 | | 0.623 | | 0.710 | |
| ST1 vs Whisper-ST1 (Ablation 5) |  | 66 | 0.323 | 0.177 |  | | 9 | | 0.298 | | 0.169 | |
| ST2 vs Whisper-ST1 (Full) |  | 66 | 0.734 | 0.734 |  | | 9 | | 0.878 | | 0.878 | |
| ST2 vs Whisper-ST1 (Ablation 1) |  | 66 | 0.381 | 0.371 |  | | 9 | | 0.638 | | 0.630 | |
| ST2 vs Whisper-ST1 (Ablation 2) |  | 66 | 0.532 | 0.558 |  | | 9 | | 0.694 | | 0.694 | |
| ST2 vs Whisper-ST1 (Ablation 3) |  | 66 | 0.420 | 0.347 |  | | 9 | | 0.613 | | 0.429 | |
| ST2 vs Whisper-ST1 (Ablation 4) |  | 66 | 0.429 | 0.409 |  | | 9 | | 0.613 | | 0.566 | |
| ST2 vs Whisper-ST1 (Ablation 5) |  | 66 | 0.393 | 0.271 |  | | 9 | | 0.439 | | 0.296 | |
| Whisper-ST1 (Full) vs Ground Truth |  | 66 | 0.789 | 0.796 |  | | 9 | | 0.893 | | 0.877 | |
| Whisper-ST1 (Ablation 1) vs Ground Truth |  | 66 | 0.505 | 0.518 |  | | 9 | | 0.689 | | 0.735 | |
| Whisper-ST1 (Ablation 2) vs Ground Truth |  | 66 | 0.641 | 0.688 |  | | 9 | | 0.780 | | 0.809 | |
| Whisper-ST1 (Ablation 3) vs Ground Truth |  | 66 | 0.476 | 0.450 |  | | 9 | | 0.623 | | 0.500 | |
| Whisper-ST1 (Ablation 4) vs Ground Truth |  | 66 | 0.500 | 0.524 |  | | 9 | | 0.623 | | 0.657 | |
| Whisper-ST1 (Ablation 5) vs Ground Truth |  | 66 | 0.379 | 0.241 |  | | 9 | | 0.347 | | 0.208 | |
